# Communication as a Tool for Exhibiting Prosocial Behavior in Dogs

**DOI:** 10.3390/ani14213091

**Published:** 2024-10-26

**Authors:** Carolina Generoso, Briseida Resende, Natalia Albuquerque, Michaella P. Andrade, Carine Savalli

**Affiliations:** 1 Department of Experimental Psychology, Institute of Psychology, University of São Paulo, Avenida Professor Mello Moraes 1721, São Paulo 05508-030, São Paulo, Brazil; carolinawood@usp.br (C.G.);; 2 Graduate Program in Evolution and Diversity, Federal University of ABC, Av. dos Estados, 5001, Bairro Bangu, Santo André 09210-580, São Paulo, Brazil; 3 Department of Public Policies and Collective Health, Federal University of São Paulo, Rua Silva Jardim 136, Santos 11015-020, São Paulo, Brazil

**Keywords:** communication, dogs, empathy, social cognition

## Abstract

Studies suggest that dogs exhibit prosocial behaviors toward humans and can rescue their guardians trapped in a box. We investigated whether dogs could communicate with an experimenter to gain access to a crying actor (the crying condition) who was visible but inaccessible, as she remained behind a fence throughout the test, or when she was simply speaking (the neutral condition). This experimenter was positioned to allow access to the actor, and the dog was free to move within the environment. Our findings indicate that dogs alternated their gazes more frequently between the actor and the experimenter, as well as between the actor and the dog’s guardian when the inaccessible actor was crying. Furthermore, the number of gazes at the guardian and the actor, as well as the duration of gazing at the actor, were greater in the crying condition. Dogs also remained closer to the actor during the crying condition. Our results suggest the dog made more communicative signals in the crying condition, bringing to light the possibility that this communication was being used as a tool to exhibit prosocial behaviors toward the actor.

## Simple Summary

There is evidence that dogs can open a door to gain access to their crying guardian, but it is unclear whether they can communicate with a person to approach another crying individual. In this study, we investigated whether dogs attempt to communicate with an experimenter to request help in accessing a crying individual (an actor) who is inaccessible. We also explored whether this communication differs if the actor offers affection to the dog prior to this experiment. Seventy-nine dogs participated in this research. Our findings indicate that dogs exhibited more visual communicative signals when the actor was speaking and crying compared to when she was simply speaking on the phone. Additionally, when the actor had previously interacted with them through play and petting, the dogs showed increased communicative behavior compared to when she did not interact. Dogs also positioned themselves closer to the actor when she was crying. The possibility of dogs communicating in a comforting context has significant implications for our understanding of empathy, communication, and animal cognition.

## 1. Introduction

Prosocial behaviors are actions aimed at benefiting another individual, and some of these actions may be driven by mechanisms of empathy [1]. In the canine literature, researchers have been exploring the extent to which dogs exhibit prosocial behaviors motivated by empathy, given their ability to discriminate certain human emotions (e.g., [2]). Evidence of various empathic phenomena in dogs includes mechanisms such as emotional contagion [3,4,5,6], mimicry [7], contagious yawning [8,9] (see [10] for a contrary perspective), and consolation [11]. For example, dogs show more physiological and behavioral signs of alertness and stress in response to human crying vocalizations compared to acoustically similar neutral sounds [3,5,6]. These findings are associated with emotional contagion, characterized by the alignment of emotional states between the individual and the observer [5]. A recent study demonstrated that dogs exhibited more stress reactions to a high-arousal, negatively valenced human sound than pigs, suggesting that selection for cooperation in dogs plays an important role in the emergence of interspecific emotional contagion [4].

Custance and Mayer [11] developed a paradigm to study empathy in dogs. They studied dog’s behavior of offering solace to humans who are expressing sadness. Their study was conducted in the dog’s home, with an experimenter and a guardian present. While the guardian or the experimenter hummed, conversed, or cried, the interactions between the dog, guardian, and experimenter were evaluated. Dogs responded differently to the guardian or experimenter when they were crying compared to the speaking and humming conditions, regardless of whether the crying agent was the guardian or an unknown person. Dogs approached, looked at, and touched the individual more during the crying condition than in the other conditions. In 2020, Meyers-Manor and Botten [12] replicated and expanded upon Custance and Mayer’s study by substituting the humming condition with a laughing condition, which is more acoustically comparable to crying and represents a different emotional state. The results were similar: dogs exhibited more person-oriented behaviors during the crying condition than during the speaking or laughing conditions. The authors suggested that this behavior indicates that dogs offer comfort to those who are suffering.

Rescue behavior is also employed to study empathy in dogs. It is a form of prosocial behavior characterized by providing help (often at a cost) to an individual in distress without an immediate reward for the rescuer [13]. This behavior is typically tested through a paradigm that involves placing the subject in a situation where they must release someone (usually a conspecific) from a distressing scenario [14]. This paradigm has been tested in ants [15,16], rats [1,17], and wild boars [18] and has been adapted for dogs by creating a situation in which the guardian was trapped behind a door displaying stress behaviors (e.g., crying), allowing the dog to open the door and release the guardian [19,20,21,22]. Overall, the results from the rescue studies in dogs suggest that they tend to open the door more frequently (or more quickly) in distressed conditions compared to neutral ones.

A recent study examined dogs’ empathic responses to unknown humans in a similar rescue paradigm [23]. The findings indicated that the distress condition of the unknown individual did not significantly affect the dogs’ door-opening behavior. The authors posited that dogs may be less inclined to display empathetic behaviors toward unknown humans compared to their guardians. This aligns with the familiar bias previously reported in empathy studies involving humans and other animals [24,25]. According to this bias, empathy is generally stronger for individuals with whom one has a closer relationship or familiarity. However, the results of this study are inconclusive, as some variables may have affected the dog’s behavior. For example, the absence of the guardian in the experimental environment may have stressed the dog and made it less likely to explore the environment [26] and display empathetic behaviors, given that dogs exhibited significantly more stress behaviors during baseline (shortly after the guardian in this experiment left after leaving of the experimental environment guardian) than during the neutral and distress condition tests. Furthermore, to mitigate the influence of fear, it would be crucial to select a sample of dogs that are comfortable with new people and unfamiliar environments. Finally, the fact that the dog encountered the person crying only during the testing phase may have heightened their anxiety, as this individual could be perceived as a threat due to their unfamiliarity in a context characterized by negative emotional valence. Given these limitations and considering that other studies have found evidence of empathic behaviors directed toward unfamiliar individuals [11], further research is needed to better understand the extent to which dogs can extend empathic behaviors to individuals other than their guardians.

In the present study, we aimed to investigate whether dogs could use communication as a tool for prosocial behavior directed toward an unknown person in an adapted rescue paradigm. Alternating gazes between a human and an inaccessible object, such as food or a toy, is a common communicative signal used by dogs when they seek access to a desirable object [27]. We questioned whether dogs would communicate to access something in the environment that is not solely linked to their own interests, as is the case of a rescue or a request for consolation.

We investigated whether dogs could communicate with the experimenter to approach a crying actor (the emotional condition) who was inaccessible/trapped (i.e., separated by a fence). The dogs’ communicative behaviors were compared in two conditions: when the inaccessible person was in distress (i.e., crying on the phone) and when she was in a neutral emotional state (i.e., simply speaking on the phone). We hypothesized that communicative behaviors toward the actor and the experimenter, such as gaze alternation and gazes, would be more frequent in the crying condition compared to the speaking condition. Additionally, we analyzed whether a prior interaction with the trapped person, which could be affective (in which the actor provided affection and attention to the dog for 10 min) or neutral (where the actor remained next to the dog on her cell phone for 10 min), would influence the dog’s behavior. Since empathic behaviors tend to be more common toward individuals with whom there is an emotional connection [25], we hypothesized that an affective interaction with the actor prior to this experiment would increase the display of communicative behaviors. Finally, we also investigated whether a demonstration by the experimenter opening the fence to give the dog access to the actor before this experiment would influence the dog’s exhibition of communicative behaviors in two conditions (crying and speaking). We hypothesized that this demonstration would enhance the display of communicative behaviors.

## 2. Materials and Methods

### 2.1. Subjects and Ethical Approval

The 109 adult dogs that participated in this experiment were recruited through the volunteer database of the Dog Laboratory at the Institute of Psychology, University of São Paulo (IP/USP), Brazil, and via social media. The final sample consisted of 79 dogs (see Supplementary Materials for background information on these dogs in Table S1) after excluding participants due to technical issues (e.g., defective camera or video; conversion problems) and procedural problems (we lost several dogs whose hair covered their eyes, making it impossible to code communicative behaviors).

Guardians completed an online form about their dogs. From this form, we selected specific questions for this study: sex, breed, size, and age. The inclusion criteria were the following: the dog had to be between 1 and 10 years old, had lived in a familiar environment for at least six months, and felt comfortable in new environments and with unfamiliar people. Visual, auditory, neurological, or chronic issues were used as exclusion criteria. This research was approved by the Committee on Ethics in the Use of Animals of IP/USP (6793130519).

### 2.2. Experimental Design and Study Location

**Figure 1:**
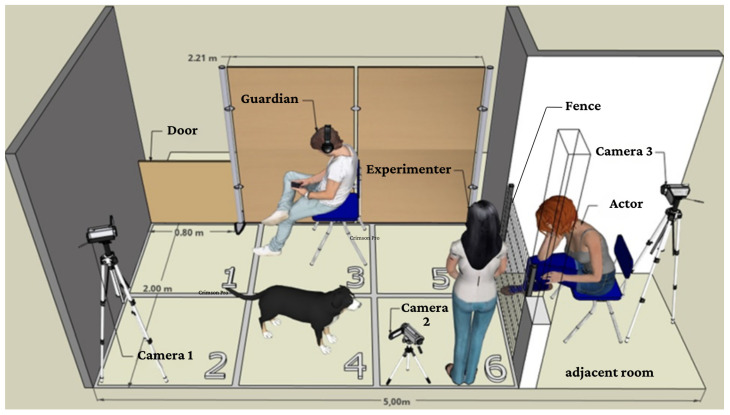
Schematic representation of the test environment with measurements. Source: Cabral, F. G. S. (2020). Made with SketchUp Free (Trimble Inc., São Paulo, Brazil). For each quadrant separated with tape, a number from 1 to 6 was assigned to delimit the dog’s location.

Dogs were subjected to a scenario involving an actor trained to cry, an experimenter (who was responsible for opening the fence), and the guardian, who was always present to minimize the stress experienced by the dog (see Figure 1 for more details about the environment). Throughout this experiment, all guardians were instructed to maintain a neutral body posture and relaxed demeanor. The guardian was told not to interact with the dog during this experiment and to appear distracted (using their phone), while the experimenter could only follow the dog’s movements with their gaze and remained silent throughout the test. The fence separating the adjacent room did not obstruct the dog’s view (Figure 1).

**Figure 2:**
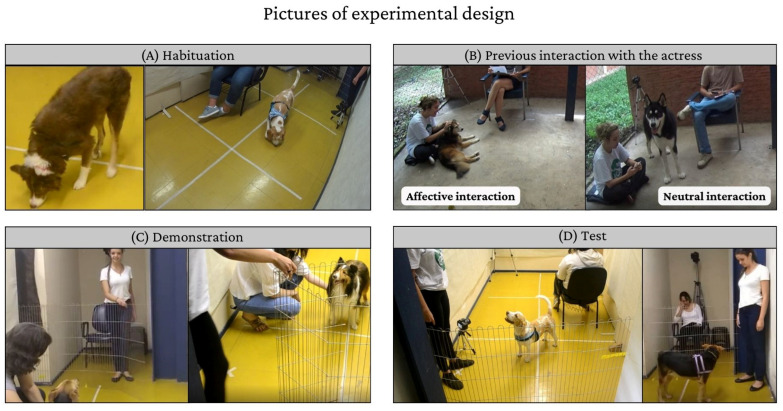
Experimental design. (**A**) Habituation: dogs exploring the environment; (**B**) Two possible interaction groups: on the left, the actor interacts with a dog in the Affective Interaction group; on the right, the actor does not interact with the dog in the Neutral Interaction group; (**C**) Demonstration; (**D**) Test: on the left, the dog is looking at the experimenter; on the right, the dog is looking at the actor.

All dogs underwent habituation, interaction with the actor, and testing phases. Half of the dogs (*n* = 40) participated in an additional phase right before the test called demonstration, which involved the experimenter opening the fence (Figure 2).Therefore, four experimental groups were formed based on the combination of the type of previous interaction with the actor (affective or neutral) and the presence of a demonstration (with or without). Dogs were randomly assigned to these four groups, and all of them experienced both conditions, crying and speaking, in a random order. The entire experiment lasted a maximum of 60 min. Upon arrival at the study site, dogs were taken to the testing room for the habituation phase. During this time, the experimenter explained the steps of this study and presented the consent form. Shortly after, guardians and dogs were directed to the open area of the Dog Laboratory for the interaction phase, and then they returned to the testing room. The group allocated to the demonstration observed the exhibition, while the other group did not.

#### 2.2.1. Habituation

Guardians and dogs underwent a habituation phase before this experiment to decrease the dogs’ interest in environmental stimuli during the test and allow them to feel comfortable in the room (duration: 5–15 min). This experimenter was attentive to any signs of discomfort or stress exhibited by the dogs. If a dog appeared uncomfortable, this experiment was halted, and the dog was excluded.

#### 2.2.2. Previous Interaction with the Actor

The interaction phase took place at the Dog Laboratory and lasted 10 min. Only the actor, guardian, and dog were present. All dogs underwent an interaction phase with the actor to standardize the contact time. For the dogs in the Affective Interaction group, the actor petted the animal (if accepted) and spoke to the dog in an affiliative manner. Twenty guardians brought their dog’s favorite toy, which the actor used during the affective interaction phase (only 10 dogs engaged with the toy; the others ignored it or played with it for less than 1 min). The use of the toy was not controlled but was permitted in cases where guardians suggested that an affective interaction could be established through play. In the Neutral Interaction group, the actor was instructed to use her mobile phone or read a book during the 10 min while the dog was free to explore the environment. The guardians remained neutral, seated in a chair while answering a questionnaire. They were instructed to maintain a neutral demeanor unless the dog displayed signs of discomfort due to the lack of attention. In such cases, the guardian was instructed to provide brief attention to avoid the dog’s frustration and then return to the questionnaire.

#### 2.2.3. Demonstration

Only dogs assigned to the demonstration group participated in this phase, which took place in the same environment as the Test phase. This phase involved demonstrating to the dog that the experimenter could open the fence and allow access to the actor. The guardian held the dog in front of the experimenter, who called the dog by name and performed the following procedure: open the fence, enter the adjacent room, exit, and close the fence (Figure 1). This procedure was repeated six times, lasting no more than one minute. Importantly, the actor was not present during this phase. The other group did not witness the demonstration and proceeded directly to the test phase.

#### 2.2.4. Test

This phase occurred in a room with four participants: a dog; a guardian; an actor; and an experimenter. At all times, the dog was free to move and could communicate (e.g., bark, alternate gaze) with all participants.

All dogs experienced two conditions, crying and speaking, in a random order. In both conditions, the actor sat in an adjacent small room behind the fence, holding a mobile phone in one hand while keeping the other motionless over her knee to avoid gestural cues. The experimenter was positioned next to the fence, always available for visual contact with the dog. The guardian sat on the other side of the room, facing away, watching a video on their mobile phone with a headset. The test commenced when the experimenter clapped her hands. The actor then simulated a telephone conversation lasting two minutes, speaking neutrally or crying according to the condition. The dialogue was identical for both emotional conditions and was presented in a different language (English) to prevent any bias from words known to the dog (Supplementary Material: Text S2). There was a 5-min interval between the two conditions.

Crying Condition: The actor was speaking (using the same script as in the neutral condition), crying, and sobbing during the phone conversation and was instructed to look slightly above the dogs’ heads and to show a sad facial expression, with corners of the mouth turned down, saddened eyes (lowered gaze), and furrowed eyebrows.

Speaking Condition: The actor simulated a telephone conversation neutrally and was instructed to look slightly above the dogs’ heads and to show a neutral facial expression.

### 2.3. Materials, Coding, and Ethogram

Three cameras were used during the test (Canon EOS Rebel SL2, Nikon D310, Sony HDR-AS200V). The videos were synchronized using the Movavi video editing program and encoded with the free Solomon Coder software (v. 17.03.22). The analyzed dog behaviors included the number of gaze alternations between the actor and the experimenter, between the actor and the guardian, and between the guardian and the door; the number and duration of gazes at the guardian, experimenter, actor, and door; as well as barking, touching the experimenter, guardian, or actor, sitting, lying down, sleeping, sniffing the environment, and sniffing the door. We also analyzed the following stress behaviors: mouth licking, yawning, crying, shaking off, scratching, and self-regulatory behaviors. The ethogram with coded behaviors during the test is provided in the Supplementary Material (Table S2). Additionally, the length of time the dog spent in each tape-marked area of the test environment was recorded. The dogs’ locations (in quadrants close to or far from the actor) were measured based on the position of the largest part of the dog’s body (mainly the head and front paws).

### 2.4. Statistical Analysis

To assess the homogeneity of the groups regarding gender, size, and breed, the chi-square test was used. In the first step, demographic factors (sex, breed, size, and age) of dogs were analyzed for all communicative behavior: the number and duration of gazes; the number of gaze alternations; and the number and duration of positions (close to the actor or far from her). Generalized Linear Mixed Models (GLMM) were applied. For the counting variables, we used Poisson distribution with a log link function. For the duration variables (positive values), we used Gamma distribution with a log link function. Dogs were included as random effects in all models. In a second step, the following experimental factors were analyzed for all response variables also with GLMM: previous interaction; demonstration and condition; and all their first and second-order (statistical) interactions. An order effect was also tested in these models. Analyzes were performed using the SAS OnDemand for Academics, and the significance level adopted was 5%.

A second observer coded, independently, the occurrences of behaviors recorded in 25% of the analyzed videos. The Kendall coefficient (W) was calculated for the agreement analysis. The following variables were scored by two observers and had good agreement: number of gaze alternations between the actor and the guardian (W = 0.73) and between the actor and the experimenter (W = 0.80); between the experimenter and the actor (W = 0.79); duration and number of times the dog positioned themselves in quadrants 1 (W = 0.84 for duration and W = 0.86 for number) and 2 (W = 0.76 for duration and W = 0.71 for number). The number of alternating gazes between the guardian and the actor presented a low agreement (below 0.70). When a third observer coded the variable, the agreement was satisfactory (W = 0.93).

## 3. Results

### 3.1. Demographic Effects

For the 79 dogs included in this experiment, the mean age was 5.02 ± 0.29 years. Of these, 53.16% were male, 44.30% were mixed breed, and 55.70% were purebred. Additionally, 32.91% were small, 41.77% medium-sized, and 25.32% large dogs. No significant differences were observed in the distribution of sex, breed, and size across the four experimental groups.

Descriptive measures of the analyzed behaviors (gaze alternation, number and duration of gazes, and dog positioning) in each experimental condition are presented in the Supplementary Material (Table S3).

For all variables regarding gaze alternation, number, and duration of gazes in all directions, no effects of sex, age, breed, or size of the dogs were found. However, an effect of age was observed on the number of times dogs remained close to the actor (F(1.78) = 4.05, *p* = 0.0477) and the number of times dogs remained far from the actor (F(1.78) = 5.34, *p* = 0.0235). Both measures decreased as the dogs’ age increased.

### 3.2. Experimental Effects

For all variables, the first and second-order (statistical) interactions among previous interaction (affective vs. neutral), demonstration (with vs. without), and condition (crying vs. speaking) were not significant (*p* > 0.05). Below, we present only the main effects found (Tables S4–S18).

#### 3.2.1. Gaze Alternation

**Figure 3:**
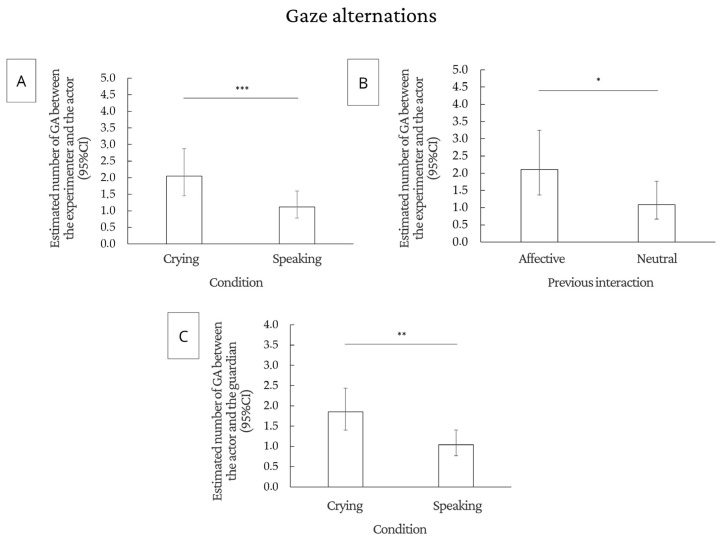
Significant results for gaze alternation (GA): (**A**) estimated number of gaze alternations between the experimenter and the actor in each experimental condition; (**B**) estimated number of gaze alternations between the experimenter and the actor in each type of previous interaction; and (**C**) estimated number of gaze alternations between the actor and the guardian in each type of condition. * *p* < 0.05; ** *p* < 0.01; *** *p* < 0.001. CI = confidence interval.

The number of gaze alternations between the actor and the experimenter was greater for the crying condition (F(1.75) = 37.6, *p* < 0.0001) and affective interaction (F(1.75) = 4.36, *p* = 0.0402), compared to the speaking condition and neutral interaction, respectively. Dogs alternated significantly more gazes between the actor and the guardian in the crying condition than in the speaking condition (F(1.75) = 27.12, *p* < 0.0001). Regarding gaze alternation between the guardian and the door, there was no effect of previous interaction, demonstration, and condition. Due to the high number of zeros (more than 60%), we did not perform inferential analysis for the data referring to the gaze alternation between the guardian and the experimenter and between the experimenter and the door. Figure 3 shows the main results regarding gaze alternation, and tables with all statistical analyses can be found in the Supplementary Material (Tables S4–S6).

#### 3.2.2. Duration and Number of Gazes

We found an effect of condition: dogs gazed more at the actor (F(1.75) = 53.24, *p* < 0.0001) and the guardian (F(1.75) = 6.36, *p* = 0. 0138) when the actor was crying compared to when she was speaking. Dogs also gazed more at the actor when the previous interaction was affective (F(1.75) = 5.15, *p* = 0.0262) compared to the neutral interaction. Regarding the duration of gazes during the crying condition, the dogs gazed at the actor longer (F(1.69) = 24.18 *p* < 0.0001). There was no effect of the prior interaction, demonstration, and condition in relation to the number and duration of gazes at the experimenter and the duration of gazes at the guardian. Finally, dogs that went through the demonstration phase looked at the door more frequently (F(1.75) = 4.25, *p* = 0.0427) compared with dogs that did not participate in the demonstration.

**Figure 4:**
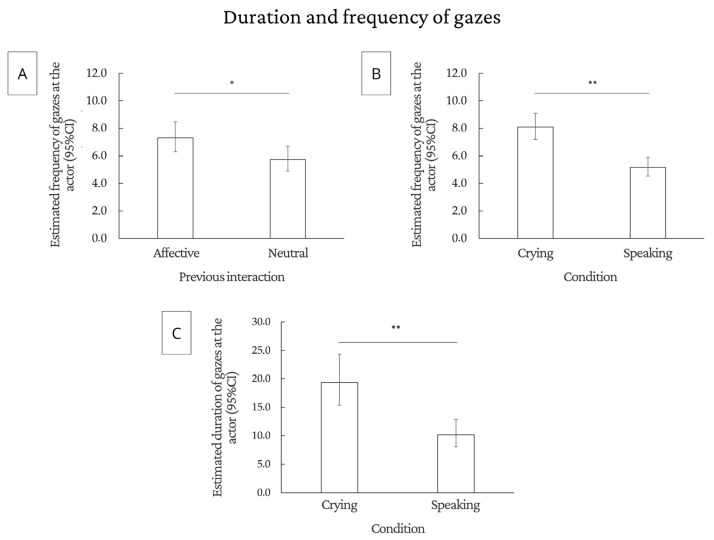
Significant results for duration and number of gazes: (**A**) estimated number of gazes at the actor in each type of previous interaction; (**B**) estimated number of gazes at the actor in each type of condition; and (**C**) estimated gaze duration for the actor in each type of condition. * *p* < 0.05; ** *p* < 0.01; CI = confidence interval.

Figure 4 shows the main results regarding the number and duration of gazes, and tables with all statistical analyses are shown in the Supplementary Material (Tables S7–S14).

#### 3.2.3. Body Positioning

**Figure 5:**
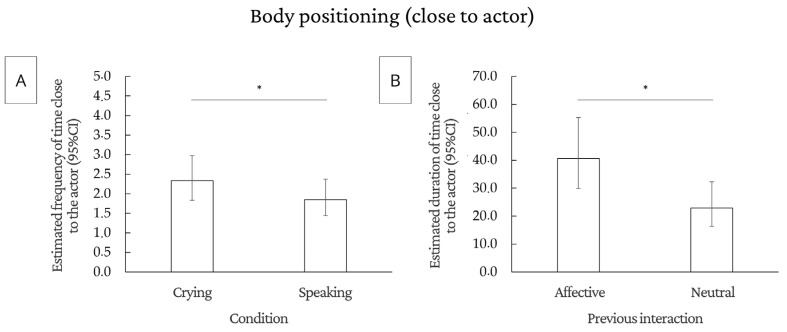
Significant results for number and duration of body positioning: (**A**) estimated number of times the dog stays close to the actor in each type of condition; (**B**) estimated duration of the dog staying close to the actor in each type of previous interaction. * *p* < 0.05; CI = confidence interval.

The number of times the dogs moved to a position close to the actor (quadrants 5 and 6) was significantly higher in the crying condition than in the speaking condition (F(1.75) = 6.06, *p* = 0.0161). The time that dogs remained close to the actor was significantly longer when the previous interaction was affective than when it was neutral (F(1.49) = 6.39, *p* = 0.0148). For both the number and duration of time that the dogs were far from the actor, there was no effect of previous interaction, demonstration, and condition (*p* > 0.05). Figure 5 shows the main results of the analyses regarding body positioning, and tables with all statistical analyses are in the Supplementary Material (Tables S15–S18).

#### 3.2.4. Other Behaviors

For the other behaviors (touching the experimenter, guardian, or actor; sitting; lying down; sleeping; sniffing the environment and sniffing the door) and for the stress behaviors (mouth licking, yawning, crying, shaking off, scratching, and self-regulatory behaviors), the descriptive measures are presented in the Supplemental Material. Inference analyses were not performed since these behaviors were rarely observed.

#### 3.2.5. Order Effect

We found an order effect for some variables: dogs that went through the “speaking and crying” order looked more at the guardian (F(1.75) = 10.65, *p* = 0.0017) and alternated more gazes between the guardian and the door (F(1.75) = 5.86, *p* = 0.0180) and between the guardian and the actor (F(1.75) = 11.43, *p* = 0.0011) when compared to dogs that went through the “crying and speaking” order. Additionally, dogs that went through the “crying and speaking” order stayed closer to the actor for longer (F(1.49) = 11.99, *p* = 0.0011).

## 4. Discussion

This study is the first to place dogs in front of a crying person in an inaccessible location and test whether they can communicate with an experimenter who can allow access to that person. Our primary results revealed that dogs alternated gazes more frequently between the actor and the experimenter, as well as between the actor and the guardian, when the actor was crying compared to when she was merely speaking. We also found that the number and duration of gazes at the actor were greater in the crying condition than in the speaking condition. Dogs gazed more at the guardian and remained closer to the actor during the crying condition. Overall, these results support our hypothesis that dogs exhibit more visual communicative signals when the actor is crying.

In previous studies, guardians themselves had to simulate crying, which can be a limitation of the demonstration [20]. To ensure the standardization and reliability of crying demonstrations and to avoid that the dog’s response had more to do with the need for social contact with the guardian than with a prosocial behavior of rescue or comfort, as in the case of the study by Sanford, Burt, and Meyers-Manor, 2018 [20], we replaced the guardian by an actor, formally trained to express the emotion of sadness and distress during crying. This experimental paradigm’s adaptation strengthens the interpretation that dogs recognize the simulated emotion rather than simply seeking to approach the guardian.

In our study, we investigated whether a dog’s response to a crying person would vary based on their previous interaction with that individual. We compared two groups of dogs: one that had an affective interaction with the actor before the test (such as play and petting) and another group that had a neutral interaction where the actor simply read a book or used her cell phone. In the studies by Custance and Mayer [11] and Meyers-Manor and Botten [12], dogs exhibited comfort-related behaviors toward both the guardian and the experimenter, indicating that they could extend possible empathic responses to unfamiliar people. Although it is likely that responses would be more pronounced toward the guardian, dogs may exhibit prosocial behaviors when previously interacting in an affective manner with the actor, given that it is known that a brief interaction with an unfamiliar person can foster an emotional bond [28]. Our results showed that the number of gaze alternations between the experimenter and the actor, as well as the number of gazes at the actor, were higher when the prior interaction was affective. Additionally, the duration of time dogs spent close to the actor was greater when the prior interaction was affective than when it was neutral. These results suggest that the bond formed and the positive emotional content transmitted during the affective interaction may have stimulated communication driven by social motivation. The increased number of gazes at the actor and the proximity of the dogs following an affective interaction indicate that she became a salient stimulus for the dogs. As we did not observe first- and second-order statistical interactions between prior interaction and condition, we concluded that one effect did not enhance the other; therefore, regardless of the crying or speaking condition, the actor became a salient stimulus because she provided relevant resources (e.g., affection, attention) for the dog.

Rivera and Meyers-Manor [23] found no differences in door-opening behavior toward an unknown person between neutral and crying conditions. In our experiment, the prior interaction with the actor and the presence of the guardian in the experimental environment may have positively influenced the absence of stress or aggressive behaviors directed toward the actor, as many dogs in the study by Rivera and Meyers-Manor [23]—especially those that did not open the door—approached the unknown person with fear and aggression. The researchers also observed a positive relationship between guardian-reported fear and latency at the start of the crying and the latency to open the door in that condition [23]. Additionally, according to the authors, the absence of the guardian during the test appeared to distress the dogs more than their inability to access the unknown person. In our experiment, we exclusively selected dogs comfortable with new people and unfamiliar environments, and we excluded those that exhibited signs of discomfort during the test, which may have facilitated the emergence of communication potentially associated with prosocial behavior since the dogs were safe and close to their attachment figure (the guardian).

Observing the experimenter open the fence did not influence the display of communicative behaviors. The lack of a demonstration effect raises two possible explanations: either the dogs did not associate the experimenter with the ability to open the door, or the demonstration was ineffective in helping the dogs understand the experimenter’s capability to open the gate. Brazilian dogs living in family homes likely encounter fences and doors throughout their lives; however, we did not collect data to account for this prior exposure. Future studies could enhance the dog’s familiarity with the apparatus, as was performed in the study by Van Bourg, Patterson, and Wynne [19]. The authors examined the dogs’ abilities to rescue their guardians from a box and evaluated whether prior experience with a box influenced the results. They found that dogs that demonstrated the ability to open the device before the test were eight times more likely to release their guardian in both stress and neutral tests. They also discovered that dogs with prior experience opening objects such as bins, cupboards, gates, or containers were four times more likely to open the device. Interestingly, dogs that underwent the demonstration phase looked at the door more frequently.

A key question in this paradigm is whether dogs exhibit emotional contagion [21]. In this context, a crying human is expected to provoke an increase in the dogs’ stress responses [4]. Stress behaviors were rarely observed in this experiment, making statistical comparisons between conditions and experimental groups impossible. In the study by Dzik et al. [22], dogs displayed more time with their ears down in conditions where the guardian was distressed compared to when they were calm, which could reflect a stress response possibly linked to contagion. However, no differences were found between neutral and crying conditions for other stress indicators (tail down, vocalizations, mouth licking). Van Bourg and colleagues [19] observed rescue behavior in dogs in scenarios where their guardian was trapped in a box, comparing two conditions: asking for help (in a state of distress) or reading (in a neutral state). They found that dogs exhibited more stress behaviors in the distress test than in the reading test, indicating that guardians transmitted their distress states to dogs through emotional contagion. Similarly, Carballo et al. [21] observed an increase in the heart rates of dogs in the distressed guardian condition but found no differences in salivary cortisol or other stress behaviors, perhaps due to the predominance of unrest over contagion. Sanford, Burt, and Meyers-Manor [20] also found no effects of dog stress on rescue behavior. In our study, we attempted to minimize the dog’s stress response by ensuring the guardian was present during the test. Thus, if stress behaviors emerged, they could potentially be linked to the actor’s crying. However, these stress behaviors were rare, likely because the presence of the calm guardian throughout the experimental conditions ensured a low level of stress in the dog, possibly providing a sense of security due to the secure base effect [26,29]. The results of these prior studies raise questions about whether the dogs’ behaviors in the present study relate to emotional contagion, given the low presence of stress behaviors in the crying condition. While some researchers view emotional contagion as a prerequisite for directed helping (e.g., rescue behavior) [30], others argue that behaviors related to comforting or directed helping can occur without necessarily involving an emotional sharing process [31]. In this case, even without contagion, dogs could communicate regarding the actor, which may involve aspects of targeted help tailored to a specific situation or need of the other, without necessarily incorporating emotional elements [32]. However, this relationship remains unclear in the literature. Future research based on the paradigm used here could include additional methods to non-invasively measure the physiological and endocrine aspects of the dogs (e.g., cortisol, heart rate, oxytocin) alongside observations of other stress behaviors to clarify this issue. Furthermore, it is worth noting that our results are limited to dogs that did not show any signs of discomfort during the test, as this was a requirement for our sample. This may have influenced the absence of stress-related behaviors.

Another important aspect to consider is that the adapted paradigm in this research does not allow for the distinction between whether the dogs’ communicative behaviors indicate a desire to rescue the crying individual or to approach them for other reasons. In either case, prosocial, consoling, or helping behaviors may be involved. In the studies by Custance and Mayer [11] and Meyers-Manor and Botten [12], dogs approached, looked at, and touched individuals in the crying condition more than in the other conditions, both for the guardian and for an unfamiliar person. Most dogs also approached in a manner considered submissive rather than playful, alert, or calm, as observed in other contexts. In our study, we questioned whether dogs, in addition to moving closer, would communicate to access the crying person. Our results indicated that when the actor was crying, dogs alternated gazes more frequently between the actor and the experimenter and between the actor and the guardian. It is well known that dogs alternate gazes when they wish to draw someone’s attention to something of interest [33], which raises the possibility that the gaze alternation in our study was related to the approach toward the actor. However, we cannot guarantee the motivations behind the behaviors observed in this study.

It could be argued that the differences found in the behaviors during the crying condition compared to the speaking condition stem from the positive reinforcement the dogs experienced throughout their lives [12]. For instance, if dogs received affection whenever they approached their guardian who was crying, it is possible that they would seek proximity to a crying individual for the same purpose. Even if these experiences were limited to their guardians or familiar individuals, it is plausible that dogs generalized their learned responses to unknown people, although this transfer of learning does not always occur in dogs (e.g., [34]).

It may also be suggested that the dogs’ communication in this context is related to seeking help to stop the distressing stimulus (crying) that troubles them, akin to personal distress [30]. That is, when experiencing the negative emotional states of others, an individual selfishly seeks to alleviate their own anguish. If this were the case, one would expect the dog to avoid looking at the actor. However, we found that the number of gazes at the actor was higher when she was crying. Furthermore, when perceiving a potentially harmful stimulus, the dogs would likely tend to seek comfort from the guardian (who was always available in the experimental room) [26] or look for an exit, which was also not observed. In fact, the number of times dogs moved closer to the actor was greater in the crying condition than in the speaking condition. If personal distress were at play, we would also expect the dogs to display stress behaviors, which our measurements did not reveal.

Our results contribute to discussions surrounding consolation and targeted helping. According to de Waal [35], consolation occurs when an animal offers comfort to a distressed partner, whereas targeted help involves the cognitive assessment of another’s situation or needs. Rescue behavior has been studied as an example of targeted help related to empathy [36]. While we cannot infer the motivations behind dogs’ communication in the context of crying or whether they intended to approach the individual for rescue or comfort, our results provide evidence for the possibility that dogs can use communication as a tool for prosocial behavior, such as targeted help or comfort.

Additionally, our findings are relevant to the literature on canine social referencing. The term “social referencing” describes a process in which an individual uses emotional information from an informant about a stimulus or object in the environment to determine their reaction toward it [34]. In the study by Merola et al. [34], dogs were exposed to a threatening object (a fan turned on) and could seek emotional information from a person present near that object. To discuss the communication related to social referencing, the experimenter or guardian would need to express some emotional content. However, since both were instructed to remain neutral throughout this experiment, and the guardian had his back turned, the dog could not extract any emotional information from a potential informant to make a decision. It is possible that at the beginning of the test, dogs sought this information from the guardian or experimenter, but we cannot conclude that the observed behaviors resulted from a process of social referencing, as guardians were instructed to remain neutral throughout this experiment.

We cannot dismiss the possibility that the acoustic characteristics of crying influenced the results; that is, the dog’s response may have been due to a more salient crying stimulus compared to the neutral one. However, in the study by Meyers-Manor and Botten [12], which measured comforting behavior, the laughing condition was acoustically comparable to the crying condition. They also found that dogs exhibited more person-oriented behaviors during the crying condition than during the speaking or laughter conditions. Another consideration regarding the dogs’ gazes at the guardian is that our recordings did not differentiate whether these gazes were directed at the guardian’s back or toward the front, which warrants further investigation in future studies, as some of these gazes may have been monitored rather than communicated.

Finally, given the order effect observed in this study, the results should be interpreted cautiously. Dogs that experienced the “speaking and crying” order gazed more at the guardian and alternated more gazes between the guardian and the door and between the guardian and the actor than dogs that underwent the “crying and speaking” order. We cannot rule out the possibility that the dog wanted to communicate with the guardian to open the door to exit the experimental environment when the crying condition occurred afterward. The fact that the speaking condition occurred first may have allowed the dog to become distracted, exploring other stimuli, making the subsequently presented crying condition more salient and capturing the dog’s attention. The longer duration of this experiment may also have heightened the dog’s desire to leave the experimental setting during the crying condition, directing more gazes toward the guardian (possibly due to fatigue). Conversely, when the crying condition was presented first, dogs may have stayed closer to the actor for longer, as she represented a more salient stimulus. Additional studies are needed to evaluate how order can influence dog behavior. Nevertheless, it is important to note that the main finding of this study is that dogs exhibited more gaze alternation between the actor and the experimenter in the crying condition. Half of the sample that received the “speaking and crying” order appeared more uncomfortable, but the absence of an order effect regarding gaze alternation between the actor and the experimenter indicates that the crying was the primary incitement for this behavior, regardless of the order in which it was presented.

## 5. Conclusions

Our study reveals a significant finding regarding the communicative abilities of dogs, specifically the alternation of gaze between the experimenter and the actor. Dogs can utilize these communicative signals in contexts that do not necessarily involve a reward, such as food or toys. Regardless of the motivation for communication in this instance (approach, help, or both), the presence of this ability provides new insights into understanding empathy, communication, and the human–dog relationship.

## Data Availability

The raw data supporting the conclusions of this article will be made available by the authors upon request.

## Supplementary Materials

The following supporting information can be downloaded at https://www.mdpi.com/article/10.3390/ani14213091/s1, Text S1: Text of the conversation simulated by the actor on the phone; Table S1: Table with basic information about the dogs tested; Table S2: Ethogram with coded behaviors and their descriptions; Table S3: Means (±Standard Error—SE) of the number of gaze alternations (logarithmic scale); of the number and duration (in seconds) of glances and body position, in each experimental condition; Table S4: Models for the number of alternating glances between the experimenter and the actor; Table S5: Models for the number of alternating glances between the actor and the guardian; Table S6: Models for the number of alternating glances between the guardian and the door; Table S7: Models for the number of gazes at the actor; Table S8: Models for the duration of gazes at the actor; Table S9: Models for the number of gazes at the experimenter; Table S10: Models for the duration of glances at the experimenter; Table S11: Models for the number of glances at the guardian; Table S12: Models for the duration of glances at the guardian; Table S13: Models for the number of glances at the door; Table S14: Models for the duration of gazes at the door; Table S15: Models for how often the dogs went to the position near the actor; Table S16: Models for duration in position near actor; Table S17: Models for how often the dogs went to the far position of the actor; Table S18: Models for Duration in Far Position of Actor. Video S1: Example of affective interaction and crying and neutral conditions.
