# Supplementary material for: Communication as a Tool for Exhibiting Prosocial Behavior in Dogs
Source: Animals (Basel). 2024 Oct 26;14(21):3091. doi: 10.3390/ani14213091 (PMC11544948; doi:10.3390/ani14213091)
Supplement: Supplementary file 1 [file animals-14-03091-s001.zip › animals-3183837-supplementary/animals-3183837-supplementary.pdf]

Supplementary material

Text S1. Text of the conversation simulated by the actor on the phone

Hi, I called to better understand what happened yesterday, because I don't know why you said that to me.

This is something that nobody talks about, and you need to improve the way you talk to people.

I think we should talk another day because we are having a hard time understanding each other.

"Goodbye".

Hi, I called to better understand what happened yesterday, because I don't know why you said that to me.

This is something that nobody talks about, and you need to improve the way you talk to people.

I think we should talk another day because we are having a hard time understanding each other.

"Goodbye".

Table S1 - Table with basic information about the dogs tested.

Dog	Sex	Age	Breed	Size
1	Male	6	Yorkshire Terrier	Small
2	Male	4	Labrador Retriever	Large
3	Female	8	Golden Retriever	Large
4	Male	5	Pug	Small
5	Male	8	Mutt	Large
6	Female	7,5	Yorkshire Terrier	Small
7	Male	3	Shetland Sheepdog	Medium
8	Male	7	Shih-tzu	Small
9	Male	6	Shetland Sheepdog	Medium
10	Male	9	Shetland Sheepdog	Medium
11	Female	1	Mutt	Medium
12	Female	2	Mutt	Medium
13	Female	6	German Spitz	Small
14	Male	6	German Spitz	Small
15	Male	3	German Spitz	Small
16	Female	7,5	Yorkshire terrier	Small
17	Female	1,5	Mutt	Medium
18	Female	4	Bull terrier	Medium

19	Male	2,5	Mutt	Medium
20	Female	7	Mutt	Medium
21	Male	1	Husky	Large
22	Female	7	Schnauzer	Small
23	Female	8	Pug	Small
24	Female	3	Staffordshire Bull Terrier	Medium
25	Male	3	Labrador	Large
26	Female	7,5	Mutt	Medium
27	Female	3	Mutt	Medium
28	Female	2	Mutt	Medium
29	Male	4	Mutt	Small
30	Male	2	Dachshund	Small
31	Male	8	Mutt	Small
32	Female	Not informed	Yorkshire	Small
33	Male	8	Golden Retriever	Large
34	Female	9	Mutt	Medium
35	Male	10	Mutt	Medium
36	Male	8	Mutt	Large
37	Male	3	Mutt	Small
38	Female	4	Mutt	Medium
39	Male	9	Bassed Hound	Medium
40	Female	6	Bassed Hound	Medium
41	Female	2	Samoieda	Medium
42	Male	2	Mutt	Small
43	Female	9	Mutt	Medium
44	Male	8	Mutt	Medium
45	Male	4	Rottweiler	Large
46	Male	5	Mutt	Large
47	Female	2	Mutt	Small
48	Female	1	Belgian Shepherd	Large
49	Male	4	Golden Retriever	Large
50	Female	2	Mutt	Small
52	Male	2	Mutt	Medium
53	Male	7	Lhasa apso	Small
54	Male	5	Mixed-breed dogs	Large
55	Female	2	Pit Bull	Medium
56	Male	4	Golden retriever	Large
57	Male	5	Border collie	Large
58	Female	4	Mutt	Medium

59	Female	4	Rottweiler	Large
60	Female	6	Mutt	Small
61	Female	7	Mutt	Small
62	Male	7	Mutt	Medium
63	Male	3	Beagle	Medium
64	Female	2	Jack Russel	Small
65	Male	4	Shetland Sheepdog	Medium
66	Male	3	Golden Retriever	Large
67	Male	7	Mutt	Small
68	Male	4	Mutt	Medium
69	Female	6	Mutt	Medium
70	Male	3	Golden Retriever	Large
71	Male	4	Shetland Sheepdog	Medium
72	Female	5	Mutt	Medium
73	Female	5	Golden Retriever	Large
74	Male	9	Mutt	Large
75	Female	1	Brazilian Terrier	Small
76	Female	10	Mutt	Medium
77	Male	8	Samoieda	Large
78	Male	2,5	Cocker spaniel	Medium
79	Female	3	Mutt	Small

Table S2. Ethogram with coded behaviors and their descriptions

Behaviors	Descriptions	Measurements
**General and communicative behaviors**		
Two-point gaze alternations:<br><br>Actor ⇌ Guardian.<br>Actor ⇌ Experimenter.<br>Guardian ⇌ Door.<br>Experimenter ⇌ Door.	The dog positions its nose/eyes towards the person/door and then towards the other person/door (or vice versa) within a maximum interval of 2 seconds.	Frequency
Gazing at guardian/actor/door/experimenter	Dog remains with nose/eyes directed towards person/door.	Frequency e Duration
Bark at the experimenter/guardian/actor	Dog makes vocal barking sound with gaze directed at person	Duration
Touching the experimenter/guardian/door	The dog touches the person/door (approximate distance equal to or less than 1 centimetre) with its head or front paws.	Duration
To sit	The dog remains sitting on the ground on its hind limbs.	Duration

To lie down	The dog remains with the thorax/abdomen or side of the body against the ground.	Duration
To sleep	The dog remains with the thorax/abdomen or side of the body leaning on the ground and with eyes closed	Duration
Sniff the environment	The dog positions the nose close to the ground (or raised) and makes quick and repeated movements of inhaling/exhaling air with the nose.	Duration
Sniff door	The dog positions its nose close to the door and makes quick and repeated movements of inhaling/exhaling air with its nose.	Duration
**Stress behaviors**		
Mouth licking	The dog protrudes its tongue through its mouth and/or nose.	Frequency
To Yawn	Movement that consists of opening the mouth at a greater angle than usual.	Frequency
To Cry	Dog makes vocal Cry sound	Duration
Shake off	The dog moves the body from side to side in a short interval	Frequency
To scratch	When the dog scratches any part of the body other than the paws.	Duration
Self-regulatory behaviors	When the dog licks or scratches its paws or sniffs/licks the urogenital region.	Duration

Table S3. Means (± Standard Error - SE) of the number of gaze alternations; of the frequency and duration (in seconds) of gazes and body position, in each experimental condition.

	**Conditions**			
**Behavior**	**Affective Interaction + With Demonstration**	**Affective Interaction + Without Demonstration**	**Neutral Interaction + With Demonstratio n**	**Neutral Interaction + Without Demonstration**
Gaze alternation between actor and experimenter	Crying: 4,90 ± 1,37 Speaking: 2,90 ± 0,49	Crying: 4,20 ± 0,83 Speaking: 2,15 ± 0,44	Crying: 2,68 ± 0,78 Speaking: 1,26 ± 0,59	Crying: 4,15 ± 1,47 Speaking: 2,60 ± 1,00
Gaze alternation between Actor and guardian	Crying: 3,20 ± 0,85 Speaking: 1,65 ± 0,44	Crying: 2,60 ± 0,60 Speaking: 2,30 ± 0,85	Crying: 3,21 ± 0,84 Speaking: 1,42 ± 0,50	Crying: 2,80 ± 0,82 Speaking: 1,40 ± 0,41
Gaze alternation between guardian and door	Crying: 5,60 ± 1,32 Speaking: 4,75 ± 1,09	Crying: 3,55 ± 1,58 Speaking: 3,20 ± 1,24	Crying: 2,58 ± 0,58 Speaking: 2,21 ± 0,66	Crying: 3,60 ± 1,28 Speaking: 3,85 ± 1,48

Gaze alternation between experimenter and door	Crying: 0,20 ± 0,12 Speaking: 0,45 ± 0,21	Crying: 0,15 ± 0,15 Speaking: 0,10 ± 0,10	Crying: 0,00 ± 0,00 Speaking: 0,05 ± 0,05	Crying: 0,00 ± 0,00 Speaking: 0,25 ± 0,25
Gaze alternation between experimenter and guardian	Crying: 1,45 ± 0,49 Speaking: 1,10 ± 0,32	Crying: 0,80 ± 0,51 Speaking: 1,40 ± 0,54	Crying: 0,68 ± 0,27 Speaking: 0,68 ± 0,35	Crying: 1,25 ± 0,55 Speaking: 1,60 ± 0,65
Gaze at the actor. (Frequency)	Crying: 9,45 ± 1,03 Speaking: 6,00 ± 0,32	Crying: 9,70 ± 0,81 Speaking: 6,60 ± 0,72	Crying: 7,68 ± 1,02 Speaking: 4,79 ± 0,81	Crying: 8,15 ± 1,26 Speaking: 5,05 ± 0,88
Gaze at the actor. (duration)	Crying: 17,96 ± 3,11 Speaking: 11,59 ± 3,25	Crying: 29,96± 5,36 Speaking: 18,51 ± 5,15	Crying: 19,67 ± 4,87 Speaking: 8,57 ± 1,96	Crying: 20,13 ± 4,61 Speaking: 9,68 ± 2,89
Gaze at the experimenter. (Frequency)	Crying: 5,00 ± 0,99 Speaking: 4,50 ± 0,87	Crying: 4,20 ± 0,64 Speaking: 4,15 ± 0,73	Crying: 3,21 ± 0,79 Speaking: 2,89 ± 0,73	Crying: 4,50 ± 0,95 Speaking: 4,35 ± 1,12
Gaze at the experimenter. (duration)	Crying: 8,22 ± 2,46 Speaking: 12,92 ± 4,24	Crying: 11,85 ± 3,37 Speaking: 13,84 ± 3,75	Crying: 7,43 ± 2,32 Speaking: 13,98 ± 5,99	Crying: 11,69 ± 3,37 Speaking: 8,98 ± 2,39
Gaze at the guardian. (Frequency)	Crying: 9,10 ± 1,40 Speaking: 7,10 ± 1,20	Crying: 7,80 ± 1,50 Speaking: 7,35 ± 1,62	Crying: 6,58 ± 0,89 Speaking: 5,32 ± 1,18	Crying: 6,95± 1,48 Speaking: 6,35 ± 1,40
Gaze at the guardian. (duration)	Crying: 14,74 ± 2,75 Speaking: 13,61 ± 3,17	Crying: 12,26 ± 2,46 Speaking: 11,59 ± 3,01	Crying: 11,43 ± 2,52 Speaking: 8,49 ± 2,63	Crying: 13,39± 3,33 Speaking: 10,73 ± 2,41
Gaze at the door. (Frequency)	Crying: 6,30 ± 1,04 Speaking: 5,90 ± 1,17	Crying: 3,90 ± 1,24 Speaking: 3,50 ± 0,89	Crying: 4,37 ± 0,95 Speaking: 3,58 ± 0,78	Crying: 3,70± 1,04 Speaking: 4,95 ± 1,39
Gaze at the door. (duration)	Crying: 19,30 ± 3,22 Speaking: 19,97 ± 5,11	Crying: 9,17 ± 2,63 Speaking: 8,98 ± 3,60	Crying: 16,94 ± 5,00 Speaking: 11,11 ± 3,75	Crying: 11,94 ± 3,89 Speaking: 14,58 ± 4,00
Positioning close to the Actor (Quadrants 1 and 2) (Frequency)	Crying: 3,55 ± 0,55 Speaking: 2,70 ± 0,55	Crying: 4,30 ± 0,79 Speaking: 3,10 ± 0,87	Crying: 2,74 ± 0,76 Speaking: 2,26 ± 0,60	Crying: 2,65± 0,80 Speaking: 2,30 ± 0,65
Positioning close to the Actor (Quadrants 1 and 2) (duration)	Crying: 39,56 ± 7,77 Speaking: 32,46 ± 8,49	Crying: 62,79 ± 8,70 Speaking: 46,85 ± 10,07	Crying: 23,45 ± 7,16 Speaking: 17,00 ± 4,91	Crying: 18,64 ± 5,39 Speaking: 28,22 ± 7,70

Distant placement of the Actor (Quadrants 5 and 6) (Frequency)	Crying: 2,60 ± 0,28<br>Speaking: 2,45 ± 0,42	Crying: 2,55 ± 0,67<br>Speaking: 2,00 ± 0,54	Crying: 2,47 ± 0,54<br>Speaking: 2,05 ± 0,45	Crying: 2,40± 0,61<br>Speaking: 2,85 ± 0,62
Distant placement of the Actor (Quadrants 5 and 6) (duration)	Crying: 53,86 ± 8,40<br>Speaking: 36,88 ± 8,94	Crying: 24,89 ± 6,19<br>Speaking: 36,97 ± 9,30	Crying: 34,75 ± 8,83<br>Speaking: 32,64 ± 8,68	Crying: 42,30± 9,47<br>Speaking: 43,64 ± 8,37

Table S4. Models for the number of gaze alternations between the experimenter and the actor

1. Results of the GLMM model for the response variable number of alternating gazes between the experimenter and the actor with demographic factors.

Effect	Test Statistics (Degrees of Freedom)	p-valor
Sex of the dog	F(1,78)=1,12	0,2931
Dog breed	F(1,78)=0,32	0,5716
Size	F(2,78)=0,06	0,9408
Age of the dog (in years)	F(1,78)=1,14	0,2879

2. Results of the GLMM model for the response variable, number of alternating gazes between the experimenter and the actor with experimental factors.

Effect	Test Statistics (Degrees of Freedom)	p-valor
Main Effect<br>**Previous interaction**	**F(1,75)=4,36**	**0,0402**
Demonstration	F(1,75)=0,04	0,8399
**Condition**	**F(1,75)=37,60**	**<0,0001**
1st Order Statistical Interaction<br>Previous interaction*Demonstration	F(1,75)=0,45	0,5050
Previous interaction*Condition	F(1,75)=0,00	0,9451
Demonstration*Condition	F(1,75)=0,13	0,7212

2st Order Statistical Interaction<br>Previous interaction*Demonstration*Condition	F(1,75)=1,20	0,2771
Order	F(1,75)=0,08	0,7810

Table S5. Models for the number of gaze alternations between the actor and the guardian

1. Results of the GLMM model for the variable response number of alternating gazes between the actor and the guardian with demographic factors.

Effect	Test Statistics (Degrees of Freedom)	p-valor
Sex of the dog	F(1,78)=0,11	0,7428
Dog breed	F(1,78)=1,25	0,2662
Size	F(2,78)=0,30	0,7448
Age of the dog (in years)	F(1,78)=1,00	0,3203

2. Results of the GLMM model for the variable response number of alternating gazes between the actor and the guardian with experimental factors.

Effect	Test Statistics (Degrees of Freedom)	p-valor
Main effects<br>Previous interaction	F(1,75)=0,86	0,3561
Demonstration	F(1,75)=0,29	0,5935
**Condition**	**F(1,75)=27,12**	**<0,0001**
1st Order Statistical Interaction<br>Previous interaction*Demonstration	F(1,75)=0,06	0,8031
Previous interaction*Condition	F(1,75)=2,70	0,1047
Demonstration*Condition	F(1,75)=2,26	0,1371
2st Order Statistical Interaction<br>Previous interaction*Demonstration*Condition	F(1,75)=0,90	0,3457
**Order**	**F(1,75)=11,43**	**0,0011**

Table S6. Models for the number of gaze alternations between the guardian and the door

1. Results of the GLMM model for the variable response number of alternating gazes between the guardian and the door with demographic factors.

Effect	Test Statistics (Degrees of Freedom)	p-valor
Sex of the dog	F(1,78)=0,09	0,7613
Dog breed	F(1,78)=3,19	0,0780
Size	F(2,78)=0,24	0,7846
Age of the dog (in years)	F(1,78)=0,43	0,5132

2. Results of the GLMM model for the variable response number of alternating gazes between the guardian and the door with experimental factors.

Effect	Test Statistics (Degrees of Freedom)	p-valor
Main effects<br>Previous interaction	F(1,75)=1,96	0,1652
Demonstration	F(1,75)=2,44	0,1228
Condition	F(1,75)=1,05	0,3087
1st Order Statistical Interaction<br>Previous interaction*Demonstration	F(1,75)=2,27	0,1360
Previous interaction*Condition	F(1,75)=0,27	0,6024
Demonstration*Condition	F(1,75)=0,66	0,4185
2st Order Statistical Interaction<br>Previous interaction*Demonstration*Condition	F(1,75)=0,21	0,6449
**Order**	**F(1,75)=5,86**	**0,0180**

Table S7. Models for the number of gazes at actor

1. Results of the GLMM model for the variable response frequency of gazes at the actor with demographic factors.

Effect	Test Statistics (Degrees of Freedom)	p-valor

Sex of the dog	F(1,78)=0,87	0,3546
Dog breed	F(1,78)=0,56	0,4582
Size	F(2,78)=0,36	0,7020
Age of the dog (in years)	F(1,78)=1,81	0,1826

2. Results of the GLMM model for the variable frequency response of gazes at the actor with experimental factors.

Effect	Test Statistics (Degrees of Freedom)	p-valor
Main effects		
**Previous interaction**	**F(1,75)=5,15**	**0,0262**
Demonstration	F(1,75)=0,23	0,6313
**Condition**	**F(1,75)=53,24**	**<0,0001**
1st Order Statistical Interaction		
Previous interaction*Demonstration	F(1,75)=0,01	0,9192
Previous interaction*Condition	F(1,75)=0,21	0,6493
Demonstration*Condition	F(1,75)=0,07	0,7971
2st Order Statistical Interaction		
Previous interaction*Demonstration*Condition	F(1,75)=0,09	0,7605
Order	F(1,75)=0,08	0,7847

Table S8. Models for the duration of gazes at the actor

1. Results of the GLMM model for the variable response duration of gazes at the actor with demographic factors.

Effect	Test Statistics (Degrees of Freedom)	p-valor
Sex of the dog	F(1,72)=3,86	0,0535
Dog breed	F(1,72)=1,55	0,2173
Size	F(2,72)=1,37	0,2616
Age of the dog (in years)	F(1,72)=0,05	0,8195

2. Results of the GLMM model for the variable response duration of gazes at the actor with experimental factors.

Effect	Test Statistics (Degrees of Freedom)	p-valor
Main Effects		
Previous Interaction	F(1,69)=1,54	0,2194
Demonstration	F(1,69)=1,51	0,2231
**Condition**	**F(1,69)=24,18**	**<0,0001**
1st Order Statistical Interaction		
Previous Interaction*Demo	F(1,69)=2,10	0,1523
Previous Interaction*Condition	F(1,69)=0,23	0,6361
Demonstration*Condition	F(1,69)=0,08	0,7718
2nd Order Statistical Interaction		
Previous Interaction*Demonstration*Condition	F(1,69)=0,67	0,4163
Order	F(1,69)=0,99	0,3240

Table S9. Models for the number of gazes at the experimenter

1. Results of the GLMM model for the variable frequency response of gazes at the experimenter with demographic factors.

Effect	Test Statistics (Degrees of Freedom)	p-valor
Sex of the dog	F(1,78)=2,58	0,1121
Dog breed	F(1,78)=0,00	0,9457
Size	F(2,78)=0,86	0,4284
Age of the dog (in years)	F(1,78)=1,18	0,2799

2. Results of the GLMM model for the variable frequency response of gazes at the experimenter with experimental factors.

Effect	Test Statistics (Degrees of Freedom)	p-valor
Main effects		

Previous interaction	F(1,75)=2,45	0,1219
Demonstration	F(1,75)=0,30	0,5867
Condition	F(1,75)=0,63	0,4072
1st Order Statistical Interaction		
Previous interaction*Demonstration	F(1,75)=0,69	0,4202
Previous interaction*Condition	F(1,75)=0,00	0,9500
Demonstration*Condition	F(1,75)=0,26	0,6117
2st Order Statistical Interaction		
Previous interaction*Demonstration*Condition	F(1,75)=0,01	0,9410
Order	F(1,75)=0,10	0,7529

Table S10. Models for the duration of gazes at the experimenter

1. Results of the GLMM model for the variable response duration of gazes at the experimenter with demographic factors.

Effect	Test Statistics (Degrees of Freedom)	p-valor
Sex of the dog	F(1,52)=2,31	0,1344
Dog breed	F(1,52)=0,49	0,4863
Size	F(2,52)=0,97	0,3856
Age of the dog (in years)	F(1,52)=0,06	0,8003

2. Results of the GLMM model for the variable response duration of gazes at the experimenter with experimental factors.

Effect	Test Statistics (Degrees of Freedom)	p-valor
Main effects		
Previous interaction	F(1,48)=0,01	0,9371
Demonstration	F(1,48)=1,16	0,2863
Condition	F(1,48)=0,83	0,6379

1st Order Statistical Interaction<br>Previous interaction*Demonstration	F(1,48)=0,76	0,3885
Previous interaction*Condition	F(1,48)=0,11	0,7433
Demonstration*Condition	F(1,48)=1,45	0,2350
2st Order Statistical Interaction<br>Previous interaction*Demonstration*Condition	F(1,48)=0,08	0,7790
Order	F(1,48)=2,94	0,0931

Table S11. Models for the number of gazes at the guardian

1. Results of the GLMM model for the variable frequency response of gazes at the guardian with demographic factors.

Efeito	Test Statistics (Degrees of Freedom)	p-valor
Sex of the dog	F(1,78)=0,00	0,9905
Dog breed	F(1,78)=1,92	0,1699
Size	F(2,78)=0,21	0,8111
Age of the dog (in years)	F(1,78)=2,19	0,1433

2. Results of the GLMM model for the variable frequency response of gazes at the guardian with experimental factors.

Effect	Test Statistics (Degrees of Freedom)	p-valor
Main effects<br>Previous interaction	F(1,75)=2,44	0,1222
Demonstration	F(1,75)=0,48	0,4885
**Condition**	**F(1,75)=6,36**	**0,0138**
1st Order Statistical Interaction<br>Previous interaction*Demonstration	F(1,75)=0,52	0,4732
Previous interaction*Condition	F(1,75)=0,00	0,9865

Demonstration*Condition	F(1,75)=1,65	0,2023
2st Order Statistical Interaction<br>Previous interaction*Demonstration*Condition	F(1,75)=0,07	0,7866
**Order**	**F(1,75)=10,65**	**0,0017**

Table S12. Models for the duration of gazes at the guardian

1. Results of the GLMM model for the variable response duration of gazes at the guardian with demographic factors.

Effect	Test Statistics (Degrees of Freedom)	p-valor
Sex of the dog	F(1,68)=0,43	0,5159
Dog breed	F(1,68)=2,44	0,1227
Size	F(2,68)=2,38	0,0999
Age of the dog (in years)	F(1,68)=0,34	0,5632

2. Results of the GLMM model for the duration response variable of gazes at the guardian with experimental factors.

Effect	Test Statistics (Degrees of Freedom)	p-valor
Main effects<br>Previous interaction	F(1,65)=0,72	0,3993
Demonstration	F(1,65)=0,24	0,6227
Condition	F(1,65)=2,38	0,1277
1st Order Statistical Interaction<br>Previous interaction*Demonstration	F(1,65)=0,90	0,3469
Previous interaction*Condition	F(1,65)=0,09	0,7688
Demonstration*Condition	F(1,65)=0,15	0,7022
2st Order Statistical Interaction<br>Previous interaction*Demonstration*Condition	F(1,65)=0,06	0,8043
Order	F(1,65)=3,52	0,0649

Table S13. Models for the number of gazes at the door

1. Results of the GLMM model for the variable frequency response of gazes at the door with demographic factors.

Effect	Test Statistics (Degrees of Freedom)	p-valor
Sex of the dog	F(1,78)=0,67	0,4166
Dog breed	F(1,78)=1,92	0,1703
Size	F(2,78)=0,47	0,6260
Age of the dog (in years)	F(1,78)=0,30	0,5826

2. Results of the GLMM model for the variable frequency response of gazes at the door with experimental factors,

Effect	Test Statistics (Degrees of Freedom)	p-valor
Main effects<br>Previous interaction	F(1,75)=1,09	0,2990
Demonstration	F(1,75)=4,25	0,0427
Condition	F(1,75)=0,07	0,7895
1st Order Statistical Interaction<br>Previous interaction*Demonstration	F(1,75)=2,31	0,1328
Previous interaction*Condition	F(1,75)=0,75	0,3889
Demonstration*Condition	F(1,75)=2,14	0,1481
2st Order Statistical Interaction<br>Previous interaction*Demonstration*Condition	F(1,75)=3,03	0,0860
Order	F(1,75)=3,33	0,0722

Table S14. Models for the duration of gazes at the door

1. Results of the GLMM model for the variable response duration of gazes at the door with demographic factors.

Effect	Test Statistics (Degrees of Freedom)	p-valor
Sex of the dog	F(1,48)=2,02	0,1620
Dog breed	F(1,48)=0,09	0,7713
Size	F(2,48)=1,92	0,1581
Age of the dog (in years)	F(1,48)=0,17	0,6862

2. Results of the GLMM model for the variable response duration of gazes at the door with experimental factors.

Effect	Test Statistics (Degrees of Freedom)	p-valor
Main effects<br>Previous interaction	F(1,44)=0,02	0,9030
Demonstration	F(1,44)=1,68	0,2018
Condition	F(1,44)=0,14	0,7115
1st Order Statistical Interaction<br>Previous interaction*Demonstration	F(1,44)=3,65	0,0625
Previous interaction*Condition	F(1,44)=0,03	0,8556
Demonstration*Condition	F(1,44)=0,16	0,6866
2st Order Statistical Interaction<br>Previous interaction*Demonstration*Condition	F(1,44)=0,57	0,4528
Order	F(1,44)=0,74	0,3955

Table S15. Models for number of times the dog is near the actor near the actor

1. Results of the GLMM model for the variable number of times that dogs went to the position near the actor with demographic factors.

Effect	Test Statistics (Degrees of Freedom)	p-valor
Sex of the dog	F(1,78)=0,84	0,3632
Dog breed	F(1,78)=0,32	0,5741

Size	F(2,78)=0,85	0,4322
**Age of the dog (in years)**	**F(1,78)=4,05**	**0,0477**

2. GLMM Model Results for Frequency Dogs Went to Position Near Actor with Experimental Factors.

Effect	Test Statistics (Degrees of Freedom)	p-valor
Main effects		
Previous interaction	F(1,75)=3,64	0,0604
Demonstration	F(1,75)=0,03	0,8591
**Condition**	**F(1,75)=6,06**	**0,0161**
1st Order Statistical Interaction		
Previous interaction*Demonstration	F(1,75)=0,14	0,7053
Previous interaction*Condition	F(1,75)=0,51	0,4794
Demonstration*Condition	F(1,75)=0,00	0,9892
2st Order Statistical Interaction		
Previous interaction*Demonstration*Condition	F(1,75)=0,07	0,7886
Order	F(1,75)=0,19	0,6646

Table S16. Models for duration in position near actor

1. Results of the GLMM model for the variable duration in the position near the actor with demographic factors.

Effect	Test Statistics (Degrees of Freedom)	p-valor
Sex of the dog	F(1,52)=1,78	0,1885
Dog breed	F(1,52)=0,54	0,4677
Size	F(2,52)=0,19	0,8305
Age of the dog (in years)	F(1,52)=0,20	0,6562

2. GLMM Model Results for Duration in Near Actor Position with Experimental Factors.

Effect	Test Statistics (Degrees of Freedom)	p-valor
Main effects		
**Previous interaction**	**F(1,49)=6,39**	**0,0148**
Demonstration	F(1,49)=3,00	0,0897
Condition	F(1,49)=2,00	0,1640
1st Order Statistical Interaction		
Previous interaction*Demonstration	F(1,49)=1,31	0,2578
Previous interaction*Condition	F(1,49)=0,35	0,5550
Demonstration*Condition	F(1,49)=0,91	0,3454
2st Order Statistical Interaction		
Previous Interaction*Demonstration*Condition	F(1,49)=3,59	0,0641
**Order**	**F(1,49)=11,99**	**0,0011**

Table S17. Models for how often the dogs went to the far position of the actor

1. Results of the GLMM model for the variable frequency that dogs went to the position far from the actor with demographic factors.

Efeito	Test Statistics (Degrees of Freedom)	p-valor
Sex of the dog	F(1,78)=0,23	0,6312
Dog breed	F(1,78)=0,34	0,5603
Size	F(2,78)=1,18	0,3140
**Age of the dog (in years)**	**F(1,78)=5,34**	**0,0235**

2. Resultados do modelo GLMM para a frequência que os cães foram para a posição distante da atriz com fatores experimentais.

Effect	Test Statistics (Degrees of Freedom)	p-valor
Main effects		
Previous interaction	F(1,75)=0,01	0,9405
Demonstration	F(1,75)=0,28	0,5956

Condition	F(1,75)=0,59	0,4437
1st Order Statistical Interaction<br>Previous interaction*Demonstration	F(1,75)=0,97	0,3283
Previous interaction*Condition	F(1,75)=0,49	0,4870
Demonstration*Condition	F(1,75)=0,18	0,6721
2st Order Statistical Interaction<br>Previous interaction*Demonstration*Condition	F(1,75)=1,73	0,1921
Order	F(1,75)=0,31	0,5790

Table S18. Models for Duration in Far Position of Actor

1. Results of the GLMM model for the variable duration in the distant position of the actor with demographic factors.

Efeito	Test Statistics (Degrees of Freedom)	p-valor
Sex of the dog	F(1,49)=0,26	0,6121
Dog breed	F(1,49)=0,35	0,5585
Size	F(2,49)=1,12	0,3358
Age of the dog (in years)	F(1,49)=0,02	0,8831

2. GLMM Model Results for Duration at Far Position of Actor with Experimental Factors.

Effect	Test Statistics (Degrees of Freedom)	p-valor
Main effects<br>Previous interaction	F(1,46)=0,39	0,5363
Demonstration	F(1,46)=0,03	0,8664
Condition	F(1,46)=1,06	0,3090
1st Order Statistical Interaction<br>Previous interaction*Demonstration	F(1,46)=0,69	0,4096
Previous interaction*Condition	F(1,46)=0,03	0,8593
Demonstration*Condition	F(1,46)=2,74	0,1046

2st Order Statistical Interaction		
Previous interaction*Demonstration*Condition	F(1,46)=1,97	0,1667
Order	F(1,46)=0,01	0,9157
